# Gene expression correlates of facultative predation in the blow fly *Chrysomya rufifacies* (Diptera: Calliphoridae)

**DOI:** 10.1002/ece3.5413

**Published:** 2019-07-15

**Authors:** Meaghan L. Pimsler, Sing‐Hoi Sze, Sunday Saenz, Shuhua Fu, Jeffery K. Tomberlin, Aaron M. Tarone

**Affiliations:** ^1^ Department of Entomology Texas A&M University College Station Texas USA; ^2^ Department of Biological Sciences University of Alabama Tuscaloosa Alabama USA; ^3^ Department of Computer Science and Engineering Texas A&M University College Station Texas USA; ^4^ Federal Aviation Administration Federal Government Office Tulsa Oklahoma USA; ^5^ Department of Biochemistry & Biophysics Texas A&M University College Station Texas USA; ^6^ Department of Developmental Biology Washington University School of Medicine in St. Louis St. Louis Missouri USA

**Keywords:** decomposition ecology, facultative predation, insects, intraguild predation, invasive species, transcriptomics

## Abstract

Effects of intraguild predation (IGP) on omnivores and detritivores are relatively understudied when compared to work on predator guilds. Functional genetic work in IGP is even more limited, but its application can help answer a range of questions related to ultimate and proximate causes of this behavior. Here, we integrate behavioral assays and transcriptomic analysis of facultative predation in a blow fly (Diptera: Calliphoridae) to evaluate the prevalence, effect, and correlated gene expression of facultative predation by the invasive species *Chrysomya rufifacies*. Field work observing donated human cadavers indicated facultative predation by *C. rufifacies* on the native blow fly *Cochliomyia macellaria* was rare under undisturbed conditions, owing in part to spatial segregation between species. Laboratory assays under conditions of starvation showed predation had a direct fitness benefit (i.e., survival) to the predator. As a genome is not available for *C. rufifacies*, a de novo transcriptome was developed and annotated using sequence similarity to *Drosophila melanogaster*. Under a variety of assembly parameters, several genes were identified as being differentially expressed between predators and nonpredators of this species, including genes involved in cell‐to‐cell signaling, osmotic regulation, starvation responses, and dopamine regulation. Results of this work were integrated to develop a model of the processes and genetic regulation controlling facultative predation.

## 1 INTRODUCTION

Intraguild predation (IGP), in which members of the community of species sharing a resource and trophic level (a “guild”) predate upon one another, is widespread in the animal kingdom (Montserrat, Magalhães, Sabelis, Roos, & Janssen, 2008; Polis & Holt, 1992). Research suggests forces such as habitat structure, which reduces the probability of interaction between the “prey” and “predator” species, and resource availability, in which increased availability of alternative food sources reduces the incidence of predation, can contribute to the coexistence of two species in the same guild (Janssen, Sabelis, Magalhães, Montserrat, & Hammen, 2007; Montserrat et al., 2008). Researchers have shown through vegetation manipulation that spatial refuge and moderately predictable and consistent prey availability can reduce IGP frequency (Janssen et al., 2007). However, other systems are not as tractable, and IGP is difficult to study in many contexts (Vanak et al., 2013). Most IGP studies have focused on the interaction of predator species with each other rather than facultative predation within a canonically nonpredacious guild (Ingram et al., 2012; Polis, Myers, & Holt, 1989; Vanak et al., 2013). However, researchers have expanded the definition of IGP outside of purely predator guilds and demonstrated the importance of IGP in structuring biological interactions in other trophic levels (Arim & Marquet, 2004).

Genetic studies of IGP (facultative or not) are scarce and may not even focus on gene expression of the focal organism (Bampfylde & Lewis, 2007; Miller, Metcalf, & Schluter, 2015). Some promising work in the threespine stickleback evaluating the effects of IGP have identified evolutionary niche and physiological trait evolution (Ingram et al., 2012; Miller et al., 2015), including evidence of a genetic basis for behavioral and morphological trait adaptation to IGP predator presence (Miller et al., 2015). There is also evidence of plasticity in these traits, as some populations exhibited predator‐avoidance traits only in the presence of the predator (Miller et al., 2015). However, this research, like others (Walzer & Schausberger, 2013), is focused on the evolutionary effect of IGP on the prey species. Both competition and predator avoidance can affect evolution and adaptive radiation (Nosil & Crespi, 2006; Oliver, Cabelli, Dolan, Jarosik, & Bioenerg, 1994). Identification of the ultimate and proximate causes of IGP in the predators will allow researchers to develop integrated models for the effect of abiotic factors on food webs (Kondoh, 2008), species coexistence, and the evolution of adaptive traits. The application of transcriptomic approaches could be a strong first step to developing testable hypotheses related to the specific stimuli, mechanisms, and pathways that lead to this complex behavioral shift.

Limited research on IGP has been conducted in patchy ephemeral resource ecology, which deals with organisms that specialize on temporary and randomly distributed resources such as fruit and vernal ponds (Janzen, 1977). As the occurrence of these resources is unpredictable and typically short‐lived, competition between organisms is expected to be fierce (Heard, 1998). Vertebrate carrion is a tractable system for patchy ephemeral resource ecology studies as researchers can control where and when it is made available. Larval flies, especially flies (i.e., blow flies) of the family Calliphoridae (Diptera) have been extensively studied in carrion decomposition work, and these organisms have long been predicted to be under intense competitive pressures (Ullyett, 1950). Several competitive strategies are employed by these flies, including rapid development, highly sensitive sensory system for detecting and locating these resources, creating an unsuitable or unattractive environment for competitors, and IGP is an example of this last approach (Bradley & Sheppard, 1984; Denno & Cothran, 1976; Ireland & Turner, 2006).

*Chrysomya rufifacies* Macquart (Diptera: Calliphoridae) represents a unique opportunity to study the factors contributing to facultative predation within a nonpredator guild. This species, native to the Orient and Australia, is a carrion‐breeding blow fly that has been distributed globally (Baumgartner, 1993). It was first detected in the New World in Brazil in 1978 and has since spread throughout the Americas (Jirón, 1979). Though first instars feed solely on decaying animal tissues, second and third instars also engage in facultative predation and cannibalism (Chitnis, 1965; Wells & Greenberg, 1992a, 1992b) though these previous studies relied exclusively on observations under laboratory conditions. Some of the potential ecological ramifications of this predatory behavior in invasive territories include driving the local extinction of native fauna, altering attraction and colonization patterns of carrion by other blow flies (Brundage, Benbow, & Tomberlin, 2014; Spindola, Zheng, Tomberlin, & Thyssen, 2016), and changing the predation patterns of beetle species (Wells & Greenberg, 1992a). Therefore, work with *C. rufifacies* presents an opportunity to study the ecological impact and molecular regulation of a complex behavior in an invasive species.

The purpose of this study was to determine the frequency of predation, putatively identify genetic markers of predation, and integrate this information to generate a model of the process leading to predatory behavior. More explicitly, this study was designed to investigate several specific aims. The first aim was focused on predation frequency and fitness effect; (a) How frequent is predation in the field? (b) How frequent is predation in simplified laboratory conditions? and, (c) Are there any fitness benefits to predation? The second aim was to compare gene expression in predator and nonpredators. In this very distinct system, there are many opportunities to learn about the forces that shape omnivory and intraguild predation. The results of this work will help generate specific testable hypotheses regarding the proximate causes of predation, the impact of supplemental food availability, and the genetic mechanisms regulating nutritional ecology in immature insects.

## 2 MATERIALS AND METHODS

### 2.1 Field behavior observation

Observations of *C. rufifacies* on human cadaver donations were made at the Forensic Anthropology Research Facility at Texas State University in San Marcos, Texas as approved by the Institutional Biosafety Committee, Texas A&M University, College Station, Texas. Institutional Review Board (IRB) approval was not needed for this work, as no personal/identifiable information was collected. Researchers observed all human cadavers with second and third instar *C. rufifacies* and at least one other dipteran species for a period of 30 min each between 09:00 and 14:00 hr on five dates between 6 June and 8 September of 2014. Remains were permitted to lay in situ without disturbance and *C. rufifacies* observed actively predating were collected. These individuals were permitted to eclose to adulthood to determine their sex.

### 2.2 Colony maintenance

*Chrysomya rufifacies* larvae were collected from numerous carcasses in College Station, Texas between May and September of 2011 and eclosed adults were identified morphologically (Whitworth, 2006, 2010). Adult flies were released into a BugDorm 1 plastic cage (30 × 30 × 30 cm; MegaView Science) and allowed to interbreed to found the laboratory colony. The colony was provided with fresh deionized water, refined sugar ad libitum and fresh beef liver blood daily as a protein source for oogenesis. Flies were maintained at 28°C for a 16:8 light:dark (L:D) photoperiod. Voucher specimens were submitted to the Entomology Museum at Texas A&M University under voucher number 716 and 717. Under the conditions of this experiment, *C. rufifacies* achieve third instar at ~72‐hr postoviposition and begin pupating at ~160‐hr postoviposition. A locally sourced colony of the native blow fly *Cochliomyia macellaria* Fabricius established in previous work (Owings, Spiegelman, Tarone, & Tomberlin, 2014) was maintained as above as a prey species.

### 2.3 Predation assays

To collect *C. rufifacies* and *Co. macellaria* larvae known ages (third instar), colonies were allowed access to an oviposition substrate of fresh beef liver in a 32.5 ml opaque plastic cup covered with a KimWipe® (Kimberly‐Clark) moistened with deionized water for a three‐hour window. After oviposition, the eggs were placed in a Percival model I‐36LLVL Incubator (Percival Scientific) at 30°C, 75% relative humidity (RH), and a 12:12 L:D. After hatching, aliquots of 100 first instars were then transferred by paintbrush to 50 g of fresh beef liver in a 32.5 ml opaque plastic cup covered with a moistened KimWipe® in a 1.1 L canning jar with approximately 100 g playground sand and a Wype‐All® on the top to prevent escape but allow air flow. These rearing jars were then placed in a Percival model I‐36LLVL Incubator (Percival Scientific) at 30°C, 75% RH, and a 12:12 L:D photoperiod. This was repeated for three times per egg collection, with a total of three biological replicates.

Predation assays were initiated 96 hr postoviposition of *C. rufifacies*. A single predation arena consisted of one third instar *C. rufifacies* and one third instar *Co. macellaria* placed in an empty 30 ml plastic cup (30 technical replicates per replicate). Each trial also included a control set of 30 technical replicates; controls consisted of a single *C. rufifacies* from the same cohort isolated in an empty 30 ml plastic cup. The predation assays were kept in the incubator under conditions previously described for two weeks. One additional replicate without controls was also conducted.

**Figure 1:**
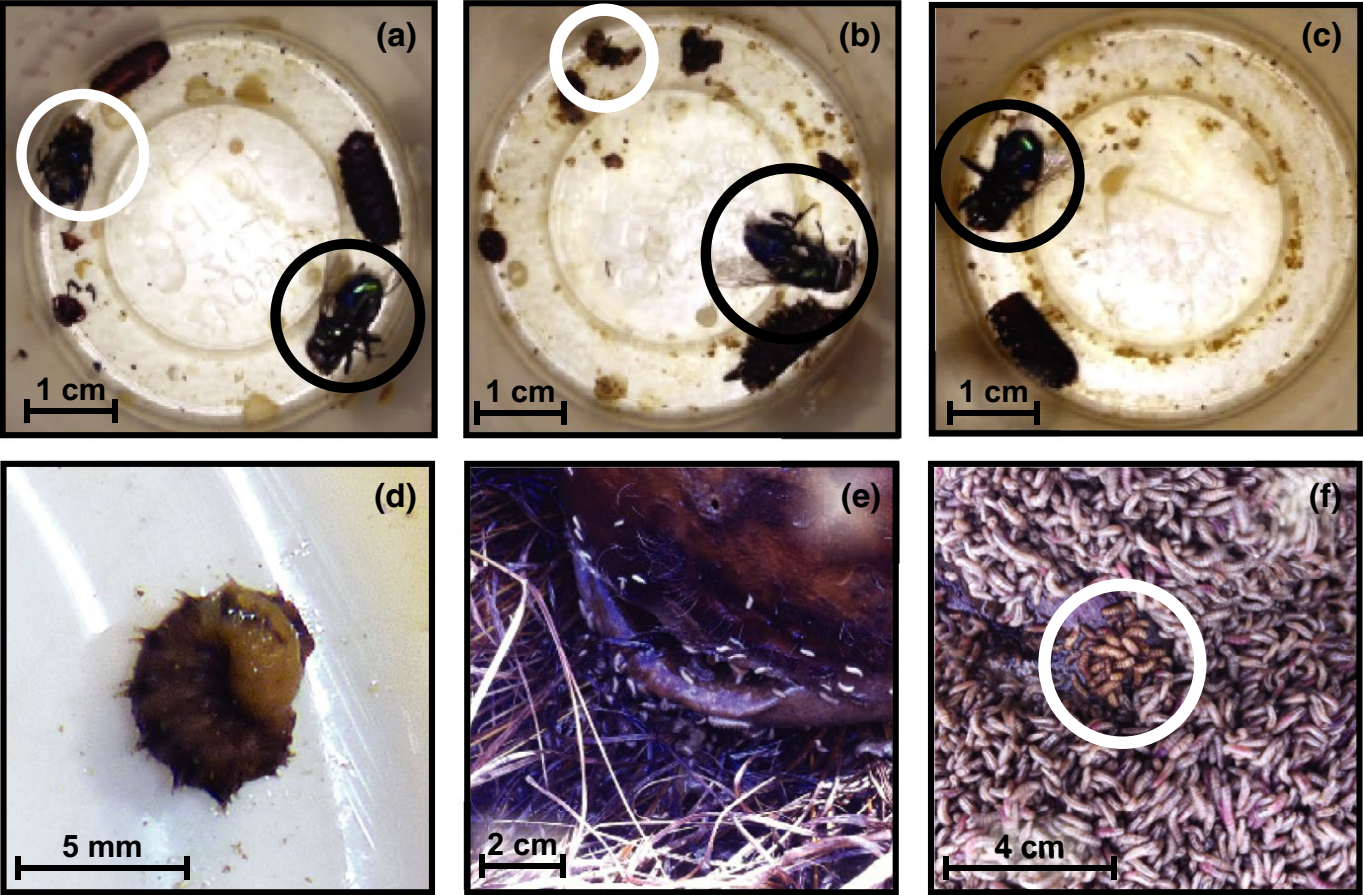
*Chrysomya rufifacies* in the laboratory and in the field, including categorization of prey consumption level of *Cochliomyia macellaria* by *C. rufifacies*. Panels (a–c) are images representative of prey consumption level categorization. Black circles indicate *C. rufifacies*, and white circles indicate *Co. macellaria*. (a) No consumption—two pupal casings (one *C. rufifacies* and one *Co. macellaria*). (b) Partial consumption—one pupal casing (*C. rufifacies*) and part of prey remaining. (c) Total consumption—one pupal casing (*C. rufifacies*) and no evidence of prey remaining. (d) When *C. rufifacies* is predating, it will wrap itself around the body of its prey item. (e) Spatial segregation between *C*. *rufifacies* and *C. macellaria* is frequently observed on human remains in the field. *Chrysomya rufifacies* is general found at the interface between the donation and the soil, with *Cochliomyia macellaria* on the surface (not pictured). (f) On the rare occasions that both species occupy the same area, the two species are found in separate masses (*C. rufifacies* in white circle)

Results were tabulated after two weeks. Each individual arena was assessed for survival of predator, sex of predator (if adult), survival of prey, sex of prey (if adult), and level of prey consumption. Prey consumption level was categorized as no consumption (i.e., whole, dead prey or prey adults eclosed), partial consumption (i.e., prey larvae partially consumed), and total consumption (i.e., prey appears absent, or the empty cuticle could be identified; Figure 1a–c). Once tabulated, data were analyzed in SAS® Studio v.9.4 (SAS Institute Inc.) to examine survival relative to supplemental food (control vs. treatment, consumption vs. nonconsumption) using Proc Freq and the Cochran–Mantel–Haenszel (CMH) test and Fisher's exact test (Mantel, 1963), as SAS is a powerful tool for categorical data analysis (Stokes, Davis, & Koch, 2012) with alpha set at 0.05.

### 2.4 Sample collection, RNA extraction, and sequencing

For each sample, a single male and female *C. rufifacies* were isolated together in a 1.1 L canning jar with approximately 100 g playground sand, a Wype‐All on the top to prevent escape but allow air flow, and refined sugar and water ad libitum and a 10 ml glass beaker filled with one KimWipe® and approximately 1 ml fresh beef liver blood. An additional 1 ml of blood was added on each following day up until the 6th day posteclosion. The protein source was then excluded for a 24‐hr period. Beginning on the 7th day of posteclosion, a 35 ml plastic cup with approximately 25 g fresh beef liver covered with a moistened KimWipe® was introduced to the jar as an oviposition medium for a four‐hour window, twice each day. If a female oviposited during this time, the females were removed, and the progeny were allowed to develop.

Approximately 96 hr postoviposition, 10 prey individuals in the third instar (*Co. macellaria*) were moved into a predation arena (10 cm diameter high walled container). After this, 10 *C. rufifacies* siblings were simultaneously moved into the predation arena. The larvae were observed until a single individual *C. rufifacies* attacked a *Co. macellaria* and exhibited the classic “wrap‐around” (Figure 1d), at which point the predator and prey were collected to an Eppendorf tube and flash frozen. Two *C. rufifacies*, which were not exhibiting predatory behavior, were also collected at the same time to control for environmental influences on gene expression. Samples were collected from a total of three maternal lines for each sex.

RNA was extracted via TriReagent preparation per manufacturer's protocols and dissolved in a 100 µl mixture of 99 µl of DNase/RNase/Nucleotide‐free water and 1 µl of SUPERase•In™ (Invitrogen, Life Technologies Incorporated). The extracted RNA was further purified using a Qiagen RNeasy Micro Kit and on‐column DNase treatment following manufacturer protocols (Qiagen Inc.). Samples were then assessed for quality and concentration using a NanoDrop 1000 (NanoDrop Products, Thermo Fisher Scientific Inc.) and Agilent 2100 Bioanalyzer Instrument (Agilent Technologies, Inc.). In total, 66 libraries across all stages of development of *C. rufifacies* (33 male and 33 female) were sequenced on four flow cells at two facilities (Sequence Read Archive BioProjects: PRJNA287123, PRJNA287124, and PRJNA385184). The 12 RNA libraries (Sequence Read Archive BioProject: PRJNA287123) that are the focus of this paper were multiplexed and sequenced with 100 bp paired‐end chemistry on two lanes of an Illumina HiSeq2500 (Illumina, Inc.) at the Texas A&M University Genomics and Bioinformatics Service. Each predator library was paired with a nonpredatory sibling library (Table 1).

**Table 1:** Sibling predator and non‐predator library numbers and abbreviations

Sex	Pair	Predator		Nonpredator	
		Library #	Sample name	Library #	Sample name
Female	1	17	P1	18	N1
	2	19	P2	20	N2
	3	21	P3	22	N3
Male	4	27	P4	28	N4
	5	29	P5	30	N5
	6	31	P6	32	N6

Note

Columns from left to right: sibling pair number (Pair), whether predator (Predator) or nonpredator (Nonpredator) sibling, library number (Library #) and name of the sample (Sample Name).

### 2.5 Transcriptome assembly and analysis

Prior to assembly, reads trimmed and processed for quality control: Reads were filtered if they contained adaptor sequences or known contaminants as defined by Illumina, including the following sequences (or their reverse complements) were removed:

“GATCGGAAGAGCACACGTCTGAACTCCAGTCACTGACCAATCTCGTATGC”, “GATCGGAAGAGCGTCGTGTAGGGAAAGAGTGTAGATCTCGGTGGTCGCCG”, “GATCGGAAGAGCACACGTCTGAACTCCAGTCACACAGTGATCTCGTATGC”, “GATCGGAAGAGCACACGTCTGAACTCCAGTCACTAGCTTATCTCGTATGC”. Finally, each read was trimmed to remove all bases including and after the first position that had a quality score of 15 or less. The 66 RNAseq libraries were sequenced with an average of 6.3 × 10^7^ reads per library at an average length (post‐trimming) of 86.3 bp; approximately 360 Gbp of sequence data was assessed. The 12 predator and nonpredator post‐trimming libraries which are the focus of this manuscript had an average of 6.6 × 10^7^ reads per library (99.96% of raw reads) at an average length of 87.4 bp and quality of 37.4; there was no difference in average size between predator and nonpredator libraries (two tailed *t* test; *p* = 0.8707).

The transcriptome was assembled with read data from all life stages of *C. rufifacies* following Sze, Pimsler, Tomberlin, Jones, and Tarone (2017) under a variety of k‐mer length (*k*) and k‐mer coverage cutoff (*c*) parameters, though only the libraries for the third instar predator and nonpredator individuals are considered here in the differential gene expression analysis. Briefly, for each k‐mer and coverage cutoff, the ASplice algorithm assembles reads into a single k‐mer_coverage assembly made up of splicing graphs arranged as nodes connected by edges, in a manner similar to how results are presented for SOAPdenovo2 (Luo et al., 2012). Nodes are analogous to unambiguously aligned contigs, and may represent an exon, or group of exons, transcribed together. The nodes of each transcriptome assembly were compared to *Drosophila melanogaster* Meigen (Diptera: Drosophilidae) proteins using the blastx algorithm (Altschul, Gish, Miller, Myers, & Lipman, 1990), keeping only the top BLAST hit with an *E*‐value ≤ 10^–7^.

Analyses comparing expression only between predatory and nonpredatory third instars from were done in R v.3.3.1 using the DESeq2 v3.1 package on all assemblies following a nested approach using best practices (Love, Huber, & Anders, 2014; R Core Team, 2018). Briefly, first a likelihood ratio test was used to identify and filter nodes which were significantly differentially expressed based on relatedness but not behavior, as these nodes likely reflected environmental similarities (testing the model “Expression ~ Behavior + Sibling pair” against “Expression ~ Behavior”). The remaining nodes were then analyzed to identify those nodes with significant differential expression between actively predating and nonpredating individuals. Sex was not included in analysis of predator gene expression as both sexes demonstrated the phenotype. Only those nodes with a false discovery rate (FDR) < 0.1 were considered for further analysis. The classes of genes identified through this analysis were predator biased and nonpredator biased nodes. The node information was analyzed relative to the assemblies to determine the number of genes, and number of transcripts of those genes that were identified as differentially expressed in each assembly. If no *D. melanogaster* hits were associated with the splicing graphs, transcripts were predicted from the splicing graphs and compared against the nucleotide and protein databases, using four different algorithms at the NCBI website: blastn (nucleotide to nucleotide database), blastx (translated nucleotide to protein database), and tblastx (translated nucleotide to translated nucleotide database) (Altschul et al., 1990) all against the nonredundant databases and the conserved domain database (CDD; Marchler‐Bauer et al., 2014). Only hits with an *E*‐value of 10^–7^ or less were considered significantly differentially expressed.

## 3 RESULTS

### 3.1 Field predation

Over the sampling period, thirteen human donations were observed to have putative *C. rufifacies* and *Co. macellaria* in at least the second instar, as this is the earliest stage at which predation has been observed in the literature (Baumgartner, 1993). Generally, the top surface of the donations was covered in *Co. macellaria* larvae, with *C. rufifacies* at the soil/donation interface (Figure 1e). Mixed masses with similar numbers of each species were not observed (Figure 1f). Interactions between *C. rufifacies* and *Co. macellaria* occurred primarily when either: *C. rufifacies* left their maggot mass and moved up onto the surface (predation was not observed in these instances), or *Co. macellaria* fell into a *C. rufifacies* mass below.

Predation events were only observed on a single donation in (D45‐2014), and only 10 larvae were observed to predate in the half hour observation window. These larvae were collected from the maggot mass under the head, though there were larvae under the whole body and observations were also made at the genital region and the left foot. Of these 10 predating larvae, eight were male. The population of *C. rufifacies* from this donation was 51% male—a sex ratio that was statistically indistinguishable from 0.5. However, the sex ratio of the predators was statistically significantly higher than 0.5 (*p* = 0.0289).

### 3.2 Behavior assays

**Figure 2:**
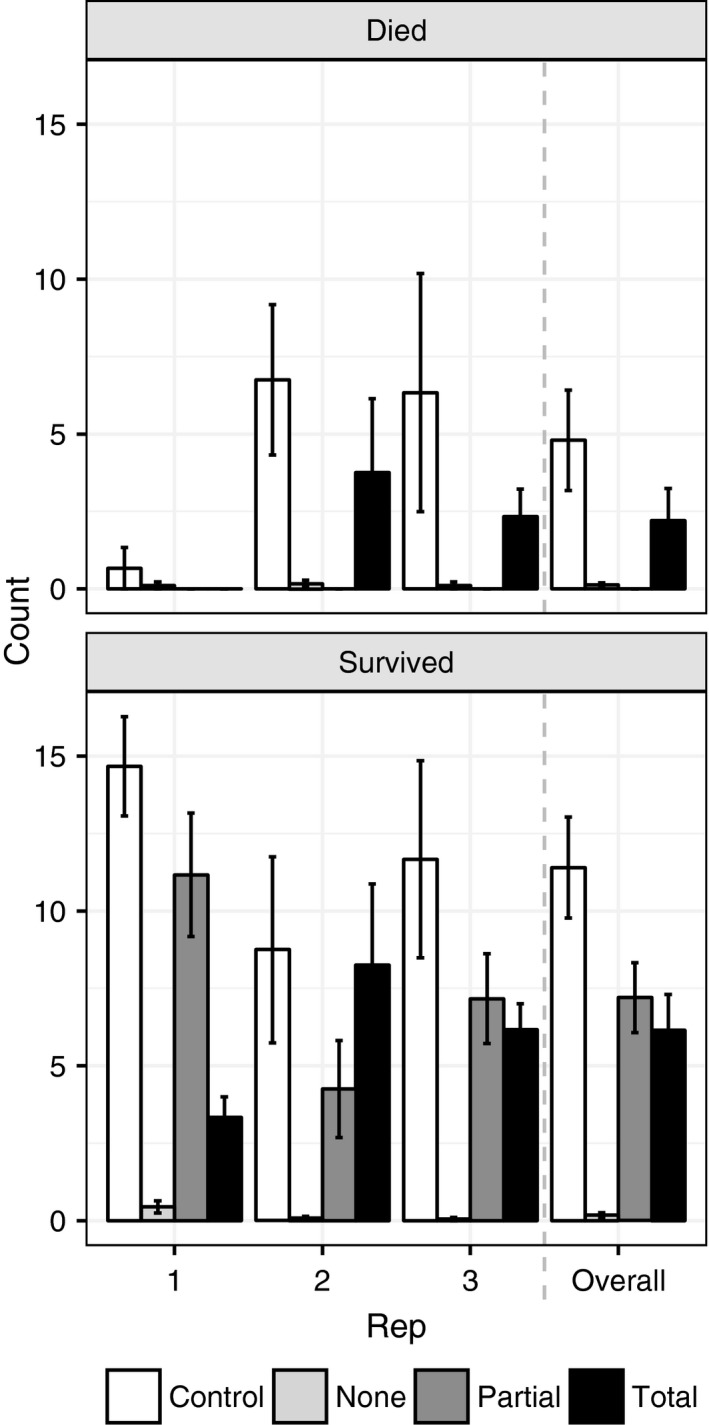
Comparison of survival rates of relative levels of predation by *Chrysomya rufifacies* on *Cochliomyia macellaria* prey in laboratory choice/no‐choice predation assays. Barplot of total number of *C. rufifacies* larvae which died (top panel) or survived to pupation (bottom panel) for each relative predation class (None, Partial, and Total from light gray to dark gray, respectively) with standard error bars of the three biological replicates with a control (no‐choice siblings, white bars), and the overall counts of survival (right of dashed gray line)

Given the difficulty of field observation, a series of laboratory experiments were conducted to quantify predation rates, feeding intensity, and investigate potential fitness benefit to predation. A total of 276 control (individual *C. rufifacies* in a plastic cup) and 304 predation (*C. rufifacies* from the same cohorts as their respective controls in a cup with access only to *Co. macellaria* larvae as supplemental food) assays were conducted over the course of three replicates (generations; Table 2). Overall survival of *C. rufifacies* was 88% with a slight but significant skew of 56% males (*Z* test on proportions, *p* = 0.0007). Consumption of supplementary food was associated with a 0.342 increased in odds of survival (CMH, *p* < 0.0001), with all individuals that engaged in partial predation surviving (Table 2, Figure 2). The sex ratios of surviving *C. rufifacies* adults in the predation assays did not differ from those of the controls (*p* = 0.3697).

**Table 2:** Predation increases survival of *Chrysomya rufifacies* in laboratory no‐choice assays

Trial	*N*_C_ (%Surv)	*N*_T_ (%Surv)	*p* ^SurvCvT^	*p* ^SurvSup^	*p* ^SurvPredLevel^
1	90 (98%)	96 (99%)	0.6111	0.2491	0.0938
2	89 (79%)	89 (91%)	**0.0352**	**0.0118**	**0.0034**
3	97 (72%)	119 (86%)	**0.0173**	**0.0063**	**0.0013**
Cochran–Mantel–Haenszel^a^	–	–	**0.0007**	**<0.0001**	0.1722
Breslow‐Day^b^	–	–	0.9501	0.5996	–
Overall^c^	276	304	**0.0017**	**0.0003**	**<0.0001**

Note

Columns from left to right: Trial, number of individuals in control group (isolated *C. rufifacies*: *N*_C_) with percent survival indicated parenthetically, number of individuals in treatment group (single *C. rufifacies* with *Cochliomyia macellaria*: *N*_T_) with percent survival indicated parenthetically, percent of *C. rufifacies* surviving to eclosion (%Surv), *p*‐value of Fisher's exact test comparing survival rates between control and treatment (Surv_CvT_), *p*‐value of Fisher's exact test evaluating the effect of supplemental food comparing survival rates between consumption (Partial and Total) and no consumption (Control and None) (Surv_Sup_), and *p*‐value of Fisher's exact test comparing survival rates by Predation level in treatment groups only (Surv_PredLevel_). Cells with a—indicate values which were not calculated because they were unnecessary or could not be calculated due to lacking data or a mathematical inability to calculate values. *p*‐Values in bold are those *p*‐values which are significant at an α = 0.05.

a Cochran–Mantel–Haenszel test for repeated tests of independence in which small *p*‐values indicate that there are significant differences between trials.

b Breslow‐Day test for homogeneity of variances where a high *p*‐value means that there is no statistically significant difference between replicates in variance.

c All of the trials collapsed and analyzed together for overall patterns.

### 3.3 Differential expression

**Figure 3:**
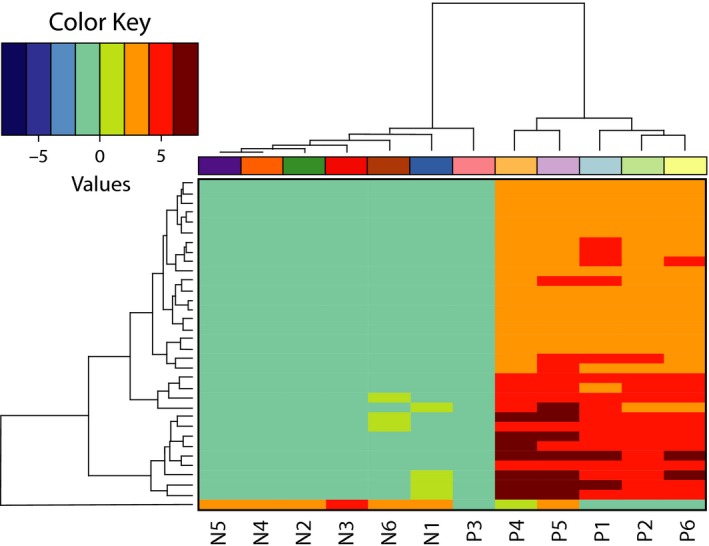
Heatmap of differentially expressed nodes between currently predating and nonpredating third instar *Chrysomya rufifacies* larvae. This figure shows a heatmap of differentially expressed nodes (rows) in 12 different libraries (columns) of currently predating individual larvae and their nonpredating siblings (same color shade, different intensity), reciprocally hierarchically clustered by similarity in expression pattern

In order to identify molecular support for hypothesized causes of predation, 24 de novo transcriptome assemblies that varied in stringency were used to evaluate the experiment (Appendix S1). On average, 18.5 nodes (maximum 34, minimum 0) in nine splicing graphs (maximum 16, minimum 0) were differentially expressed between predators and nonpredators (Table 1). These differentially expressed nodes were predominantly upregulated in predators, relative to their nonpredatory siblings (Table 3, Figure 3). One female predator did not show the same pattern of gene expression as the other predator libraries (Figure 3). Some of these splicing graphs lacked homology to any known *D. melanogaster* genes, and some were found to align to multiple genes. A total of 36 genes were annotated, with most (23) only being detected once in four or fewer assemblies. Of the remaining 13 genes (Table 3), most were detected only once per assembly‐ the exception was *AMP‐activated protein kinase α subunit* (*CrAMPKα*), detected in two different splicing graphs in both 25_50 and 25_100 assemblies.

**Table 3:** Genes differentially expressed between predators and nonpredators in de novo* Chrysomya rufifacies* transcriptomes

	No.	Name	Experimental function	Predicted function	Effects in *Drosophila*
↑	20	*Host cell factor*	Chromatin binding; contributes to histone acetyltransferase activity	Sequence‐specific DNA binding transcription factor activity; transcription coactivator activity	activation and the regulation of cellular growth (Furrer et al., 2010; Mahajan, Johnson, & Wilson, 2003). component of regulatory networks related to osmotic stress (Suganuma et al., 2010)
↑	20	*Arginase*		Arginase activity; metal ion binding	catabolize arginine in *Drosophila* (*arg*) (Sardiello, Licciulli, Catalano, Attimonelli, & Caggese, 2003) putative genetic marker of aggressive *Drosophila* (Edwards, Rollmann, Morgan, & Mackay, 2006)
↑	18	*AMP‐activated protein kinase α subunit*	AMP‐activated protein kinase activity	ATP binding; G protein‐coupled receptor kinase activity; protein serine/threonine kinase activity	part of the *Target of Rapamycin* (*TOR*) signaling pathway (Bland et al., 2010; Stroschein‐Stevenson et al., 2006) nutrition‐state dependent behavior and physiology (fat body signaling, smooth muscle function, nutrient absorption; Bland et al., 2010; Johnson et al., 2010; Stroschein‐Stevenson et al., 2006) role in dendrite morphogenesis (Swick et al., 2013)
↑	16	*Silver*	Carboxypeptidase activity; metallocarboxypeptidase activity	Metallocarboxypeptidase activity; serine‐type carboxypeptidase activity; zinc ion binding	contains carboxypeptidase D (CPD) domain (Sidyelyeva & Fricker, 2002), preference for C‐terminal arginase residues (Sidyelyeva, Baker, & Fricker, 2006) expressed in trans‐Golgi network (Varlamov & Fricker, 1998) involved with secretory protein processing (Novikova et al., 1999; Song & Fricker, 1995; Varlamov & Fricker, 1998), specifically the dopaminergic pathway; mutants have increased N‐acetyldopamine levels (Walter et al., 1996)
↑	14	*Transport and Golgi organization 5*			associated with endoplasmic reticulum and Golgi apparati (Bard et al., 2006), conserved across kingdoms (Rabouille & Kondylis, 2006; Wang et al., 2011) required for protein secretion (Rabouille & Kondylis, 2006) expressed in the central nervous system of immature Diptera (dos Santos et al., 2015)
↓	11	*Asterix*			classically known for role in gametogenesis (Dönertas et al., 2013) required for Piwi‐piRNA (Piwi‐interacting RNA) complex silencing of transposons in the female germ line (Dönertas et al., 2013) highest expression in larvae localized to fat body (dos Santos et al., 2015)
↑	10	*Glass bottom boat*		Growth factor activity; transforming growth factor beta receptor binding	part of the *Bone Morphogenetic Pathway* (*BMP*; Akiyama et al., 2012; Ballard, Jarolimova, & Wharton, 2010) nutrition‐state dependent behavior and physiology; sensitivity to nutritionally available lipids (Ballard et al., 2010) neuron morphogenesis and proper function and morphology of the neuromuscular junction (Akiyama et al., 2012; Keshishian & Kim, 2004; McCabe et al., 2003)

Note

This table summarizes the results of analysis of genes differentially expressed between actively predating and nonpredating *C. rufifacies* third instars across 24 de novo transcriptome assemblies limited to genes differentially expressed in at least 10 different assemblies. Columns (left to right): Upregulated (up arrows) or downregulated (down arrows) in predators, number of assemblies detected in (#), name of gene based on *Drosophila melanogaster* annotation (Name), experimental molecular function(s) (Experimental), predicted molecular function(s) (Predicted), and known effects and interactions of this gene or its homologues in other species (Effects).

Eleven transcripts with significant homology to *D. melanogaster* genes were differentially expressed in seven or more assemblies. Three have not yet been named in *D. melanogaster*, though two of these were annotated with predicted function information. DmCG5254 is predicted to have mitochondrial transmembrane transporter function, and DmCG1336 contains a calponin‐like domain. The four most frequently homologous predator upregulated genes were *Host cell factor* (*CrHcf*), *arginase* (*Crarg*), *CrAMPKα*, and *silver* (*Crsvr*) (20, 18, and 16 times respectively). Only one gene, *Asterix* (*CrArx*), was upregulated in nonpredators and detected in more than seven assemblies.

Splicing graphs without significant *D. melanogaster* homology were also differentially expressed, though most could not be annotated with existing databases. One notable exception was the differential expression of nodes in two different assemblies which both had homology to *Musca domestica* L. (Diptera: Muscidae) *Hcf* (XM_005187593.2). Some nodes also demonstrated high homology to domains not found in insects: Nematoda phylum NADH dehydrogenase subunit 2 (*E* < 10^–10^), EpsG family proteins related to a *Bacillus subtilis* (Cohn) (Bacillales: Bacillaceae) membrane‐bound glycosyl transferase (*E* < 10^–10^), Bacteriodes phylum integral membrane protein (domain of unknown function) DUF4271 (*E* < 10^–9^), and *Borellia* genus ORF‐A (also of unknown function; *E* < 10^–9^).

## 4 DISCUSSION

This study determined facultative predation of *Co. macellaria* by *C. rufifacies* was prevalent in the laboratory but proved difficult to observe under field conditions. Facultative predation under conditions of starvation in the third instar provided a direct fitness benefit. Incomplete spatial segregation between *Co. macellaria* larvae on the surface and the *C. rufifacies* at the interface between the soil and the donations reduced the frequency of encounters between predators and potential prey. The observations reported here support the hypothesis that species coexistence in this system may be due to resource partitioning (Amarasekare, 2007; Fiene, Sword, VanLaerhoven, & Tarone, 2014), as has been observed in other systems involving IGP (Vanak et al., 2013) and invasive species interactions (Coccia et al., 2016). Predation occurred frequently under laboratory conditions (92% of predation assays); however, and access to supplementary food resulted in increased survival. Of note, individuals in the “partial predation” class demonstrated higher survival than either “none” or “complete” predation. This may be due to the fact that individuals which partially consumed their prey item may have been able to feed to satiation, whereas individuals that completely consumed their prey were still unable to acquire sufficient nutrition. This would be in line with research that has demonstrated that insects (including blow flies) can and do regulate feeding (Bowdan & Dethier, 1986; Söderberg, Carlsson, & Nässel, 2012), though there is evidence that food availability can affect satiation detection (Kanarek & Collier, 1983).

Taken together, the laboratory and field observations suggest two interesting hypotheses. The conflict between predation rates in the field and the laboratory may be due to the temporal scale of observations or the different experimental designs. Research has shown that many fly behaviors, from carcass attendance to aggressive behavior, demonstrate distinct temporal patterns within a day (Benelli, 2014; Ceriani et al., 2002; Mohr & Tomberlin, 2014). Therefore, one potential explanation for the low rates of predation observed on undisturbed remains in the field is this behavior may be circadian rhythm gated and observations were not made at the correct time of day. The laboratory assays, in contrast, occurred over a time scale of weeks, and would therefore not have been sensitive to time‐of‐day specific behaviors. Another explanation is the predation behavior of *C. rufifacies* may be stimulated by carcass disturbance, given that no observations of predation behavior were made in the field after sample collection, and that the laboratory experimental conditions necessitated a “disturbance.” The ability to switch food sources from a preferred resource (carrion) to an abundant alternative (other dipteran larvae) may represent a significant evolutionary advantage in systems with abundant vertebrate scavengers, as scavenging by vertebrates can be a significant factor affecting the survival and fitness of carrion‐breeding insects (DeVault, Olson, Beasley, & Rhodes, 2011). Future work should determine whether predation behavior frequency changes throughout the day and examine the effect of scavenging (real and/or simulated) on predation rates.

A relatively short list of seven genes was identified as differentially expressed between actively predating and nonpredating siblings. These results point to several specific hypotheses related to both the proximate and ultimate causes of this behavior that can be further pursued. The first is that predation may be a general response to starvation, as predators exhibited upregulation of genes related to the *TOR* signaling pathway and lipid metabolism (Bland, Lee, Magallanes, Foskett, & Birnbaum, 2010; Johnson et al., 2010; Stroschein‐Stevenson, Foley, O'Farrell, & Johnson, 2006). The second is that microbial signals may stimulate predation, as some differential expression was most closely associated with bacterial domains. The third is that predation may be a response to osmotic stress (Suganuma et al., 2010). Certainly, the enrichment of *CrHcf* in predatory individuals coupled with the results of previous work that showed that *C. rufifacies* only engages in cannibalism under conditions of water stress (Chitnis, 1965), suggests water stress cannot fully be ruled out as a cause of predation. The fourth is that there may be neurological differentiation between predatory and nonpredatory individuals. Some of the differences may be due to dendrite morphogenesis (Swick, Kazgan, Onyenwoke, & Brenman, 2013) and proper function and morphology of the neuromuscular junction, respectively (Akiyama, Marqués, & Wharton, 2012; McCabe et al., 2003), whereas others may be due to differences in dopamine synthesis or management (Varlamov & Fricker, 1998; Walter et al., 1996). Finally, small RNA regulation may provide the “brakes” on predatory behavior, as miRNA has been shown to affect behavior in other insects (Asgari, 2013). The only identifiable gene downregulated during predation is *Asterix* (*Arx*), a crucial component of some piRNA‐guided silencing in *Drosophila* (Dönertas, Sienski, & Brennecke, 2013) which may assist *argonaute‐*family/PIWI complexes in regulating gene expression (Czech & Hannon, 2016). Downregulation of this one sequence correlates with upregulation of a much larger suite of genes in predators. Interestingly, the only predatory female that did not cluster with her group by gene expression did exhibit downregulation of *CrArx*.

All of the above hypotheses can be pursued in greater detail and the work described here provides more detailed potential mechanisms that could be regulating the behaviors. Particularly in light of the fact that the molecular interpretations explored here are extrapolated from *Drosophila* and therefore require validation of function in *Chrysomya*. Future work could investigate the effects of a manipulated diet (i.e., addition of the antibiotic rapamycin, which inhibits the TOR pathway; an amino acid, such as arginine or dopamine receptor agonists or antagonists), modified microbial communities, and relative humidity on predation rates to begin to tease apart the relative contributions and roles of these factors in facultative IGP. Other studies, such as the effect of *Crasx* and a putative inhibitory regulation of predation, may require transgenic techniques to determine if proper *Crasx* function is necessary and/or sufficient to inhibit predatory behavior.

**Figure 4:**
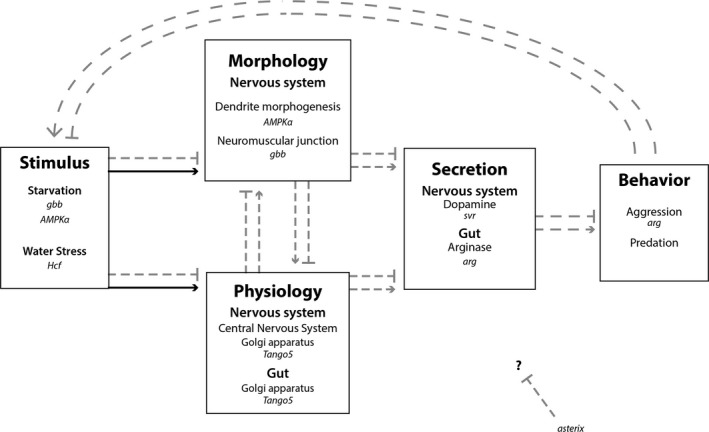
Hypothesized model for the regulation of facultative IGP in carrion‐breeding Diptera. This model is based on differential gene expression in *Chrysomya rufifacies*; any one of these specific boxes can be further investigated, as they each identify features of the system that could be tested in greater detail. Arrows indicate amplification of downstream box, bars indicate inhibition. Solid black lines indicate factors known to affect downstream modules and dashed gray lines indicate a need for empirical research

Based on our gene expression analysis, we have developed a model regarding the physiological and genetic mechanisms underlying facultative intraguild predation (illustrated in Figure 4). The process begins with a stimulus (e.g., starvation, water stress, and/or microbes) being applied to the organism and initiating physiological and functional genetic changes. Individuals with pre‐existing neurological morphologies (e.g., dendrite morphologies, in the central nervous system or at the neuromuscular junction, especially in the gut) or exposed to different environments are sensitive to this stimulus which may lead to the secretion of dopamine in the nervous system and arginase in the gut. These secretions interact with the pre‐existing morphology and physiology leading to aggressive behavior and facultative intraguild predation. Some of the genes leading to this pathway may be negatively regulated by *Crasx*.

These results seem to indicate that there is a fitness benefit to predation primarily as a response to starvation and, to a lesser extent, water stress. There are different potential levels of control to this behavior. Support from this study would suggest that physiological changes in the fly, possibly affected by microbial gene expression or circadian rhythm gating, triggers the predation behavior. The neurological route regulating the behavior in this system is likely through dopaminergic pathways.

This work also highlights the utility of transcriptomics of nonmodel organisms to develop specific testable hypotheses regarding the ultimate and proximate causes of behaviors of interest. The application of transcriptomic techniques to IGP studies will open the door for the identification of the molecular basis of complex behavior, the similarities or differences in patterns of change in genetic pathways in relation to behavioral convergent evolution, and the adaptive mechanisms leading to the trophic promiscuity of omnivores (Hunter, 2009; Tanabe & Namba, 2005). In higher order organisms with more complex behaviors this approach may not be as useful due to safety, practicality, or other concerns. However, systems such as the one studied here as well as IGP observed in omnivorous mirids (Heteroptera: Miridae) (Lucas, Fréchette, & Alomar, 2009), would be particularly tractable and possible with currently available tools. Researchers have already identified some of the environmental conditions that regulate IGP frequency in these systems (e.g., *C. rufifacies* (Baumgartner, 1993; Chitnis, 1965; Shiao & Yeh, 2008; Wells & Greenberg, 1992a, 1992b) and mirids (Lucas et al., 2009)). With this framework, identification of the molecular basis of this behavior and comparative transcriptomics will help identify the conditions that contribute to resource shift in this species. More broadly, such work will help us identify the traits in a species that make such behavioral plasticity possible, the conditions which select for the variability, and their ecological consequences.

## CONFLICT OF INTEREST

None declared.

## AUTHOR CONTRIBUTIONS

MLP helped conceive of the project, did the fieldwork, predation assays for RNA sample collection, RNA extractions, data analysis, and writing. SHS and SF did the transcriptome assembly, *Drosophila* gene annotations, and some writing. SS and MLP did the laboratory predation assays to evaluate survival, and SS provided some text for the manuscript. JKT and AMT helped conceive of the project, secure funding and contributed to writing and data analysis.

## Data Availability

DNA sequences on SRA BioProject: PRJNA287123, PRJNA287124, and PRJNA385184. Assembled transcriptome files and derivatives, and exemplar analytical R script, and raw laboratory no‐choice predation assay results—Dryad https://doi.org/10.5061/dryad.252b9n4.
